# Underpricing, underperformance and overreaction in initial public offerings: Evidence from investor attention using online searches

**DOI:** 10.1186/s40064-015-0839-4

**Published:** 2015-02-14

**Authors:** Tomas Vakrman, Ladislav Kristoufek

**Affiliations:** Institute of Economic Studies, Faculty of Social Sciences, Charles University in Prague, Opletalova 26, Prague, 110 00 Czech Republic; Warwick Business School, University of Warwick, West Midlands, Coventry, CV4 7AL UK; Institute of Information Theory and Automation, Czech Academy of Sciences, Pod Vodarenskou vezi 4, Prague, 182 08 Czech Republic

**Keywords:** Online searches, Initial public offerings, Puzzles

## Abstract

Online activity of Internet users has proven very useful in modeling various phenomena across a wide range of scientific disciplines. In our study, we focus on two stylized facts or puzzles surrounding the initial public offerings (IPOs) – the underpricing and the long-term underperformance. Using the Internet searches on Google, we proxy the investor attention before and during the day of the offering to show that the high attention IPOs have different characteristics than the low attention ones. After controlling for various effects, we show that investor attention still remains a strong component of the high initial returns (the underpricing), primarily for the high sentiment periods. Moreover, we demonstrate that the investor attention partially explains the overoptimistic market reaction and thus also a part of the long-term underperformance.

## Introduction

The Internet, a revolutionary invention from 1965 with more than two billion users by 2014, has undoubtedly changed the world we live in. It allows its users to access an unprecedented amount of information in a very short time. Due to the abundance of available information, attention has become a scarce resource that needs to be efficiently allocated in order to acquire the information of interest. For a vast majority of Internet users, search engines serve as a gateway to all that information, and Google, with its dominant market share and more than one billion unique visitors every month, is their uncrowned king. Such an online behavior leaves a digital trace. All individual search queries that have been typed into the search bar are stored by Google and the processed statistics on searches are made publicly available by the company via its Google Trends facility. The Google search volume databank thus provides a direct measure of attention which is freely available, timely and representative to the whole population of Internet users.

Such an extreme potential of Internet search data has been put into practice and it is now being used for tracking or even anticipating various social phenomena. The utilization ranges from influenza tracking (Dugas et al. 2012; Ginsberg et al. 2008), consumer interest and its impact on product sales (Choi and Varian 2009; Goel et al. 2010; Kulkarni 2012) to macroeconomic indicators (Askitas and Zimmermann 2009; Cooper et al. 2005; Preis et al. 2010). The work of Merton (1987) suggests that attention may be also relevant for the complex reality of financial markets and Preis et al. (2008) are among the first ones to support this hypothesis using the web search data to proxy attention. Since then, many researchers have used online attention to either track, nowcast or forecast various financial indicators. Here, we utilize Google searches to help us explain two stylized facts of the initial public offerings (IPOs) – the long-term underperformance and the high initial returns, also known as the IPO underpricing.

The long-term underperformance (i.e. an inferior performance to non-issuing firms) is arguably the most attractive area of the IPO academic research. Stern and Borenstein (1985) show that the issuing firms under-perform the S&P 500 index by 22% in the long-term. The underperformance has been confirmed by several studies (Ritter 1991; Spiess and Affleck-Graves 1995), most notably by Loughran and Ritter (1995) who labelled the long-term performance of the newly issued stocks as a puzzle. Its existence has been questioned by various studies. Brav *et al.* (2000) report that the underperformance disappears when the benchmarks are matched on firm size and book-to-market ratios. Conversely, Eckbo and Noril (2000) attribute the potential underperformance to a lower risk of the IPO stocks, providing evidence that the issuing companies have lower leverage ratios and higher liquidity than the matched firms in years following the IPO. After controlling for the additional risk of peer companies, the authors do not reject the hypothesis of zero abnormal returns of the IPO stocks. Ritter and Welch (2002), in their comprehensive review of the IPO related literature, argue that the benchmarking of the long-term performance of IPOs is highly sensitive to an employed methodology as well as to the choice of a sample period. In addition, they note that despite the similar (unappealing) performance of issuers and their peers with comparable characteristics, the equally weighted post-IPO returns still underperform market indices.

The existence of the second IPO stylized fact – underpricing – is rather indisputable. Ritter and Welch (2002) report that the average difference between the offer price and the first day closing price was 18.8% for the US issuers between 1980 and 2001. Furthermore, there was a positive price change for 70% of the issuing firms, while negative initial return was exhibited only by 14% of the IPOs. The reason why the issuing firms leave money on the table remains unclear here. This is further studied by Ritter and Welch (2002) who offer a wide variety of explanations based on both symmetric and asymmetric information arguments. The most promising stream of literature struggling to explain the underpricing seems to be focused on the behavioral side of investors. Ritter (1991) sheds some light on the topic by pointing out that investors tend to be periodically overoptimistic about the potential of issuing firms, and that the firms take an advantage of it by timing the offerings correspondingly. Loughran and Ritter (1995) provide some support to the hypothesis by showing the first day returns are significantly higher following the periods when the market has grown. In line with the investor sentiment theory, it has been shown that the underpricing is positively associated with news and non-lead analyst research coverage of IPOs (Aggarwal et al. 2002; Demers and Lewellen 2003).

Ljungqvist *et al.* (2006) and Derrien (2005) offer theoretical models for the IPO pricing and initial returns in the presence of investor sentiment. The former study (Ljungqvist et al. 2006) builds its model on the assumption of budget-constrained sentiment investors who cannot buy the entire IPO. Therefore, the firms must set the offer price below the level noise traders are willing to pay in order to induce rational investors to participate. The latter study (Derrien 2005), on the other hand, highlights the assumption that “*aftermarket price support is costly for the underwriter*” [(Derrien 2005) p. 490]. While the models are different in construction, their predictions are rather similar. They predict the high underpricing in presence of high investor sentiment and consequently the poor long-term performance. Derrien aptly notes that it is not the firms who leaves the money on table but rather “*the overoptimistic noise traders who pay excessive prices for IPO shares on the aftermarket*” [(Derrien 2005) p. 490].

The empirical evidence favors these models. Cook *et al.* (2006) reveal that underwriters promote IPOs in order to induce the sentiment investors into the market. It has also been reported that sentiment influences the initial pricing and that underwriters do not base their valuation solely on fundamentals and comparable valuation. The higher initial returns of IPOs that exhibited an above average abnormal attention (measured by Google search volume) and subsequent return reversal of such stocks in the long-term form the most notable empirical validation of the sentiment theories (Da et al. 2011). Here, we focus on these two IPO stylized facts in the USA between 2004 and 2010. As the measure of attention, we utilize search queries provided by Google and we examine whether such attention can be used to explain and describe the IPO underpricing and long-term underperformance.

## Methods

### 2.1 Data

#### 2.1.1 Variables construction

Studying the two stylized facts about IPOs stems in defining two types of returns – an initial return and a long-term return. We define the initial return (which we also refer to as a first day return or we abbreviate it as IR) as

(1) $$ {IR}_{i}=\log\left(P_{i}^{Close}\right)-\log\left(P_{i}^{Offer}\right),  $$

where $P_{i}^{Close}$ and $P_{i}^{Offer}$ refer to the closing price on the first day of trading and the offering price, respectively, for the IPO *i*. The long-term cumulative logarithmic return is defined as

(2) $$ {CLR}_{i}=\log\left(P_{i,t+k}^{Close}\right)-\log\left(P_{i,t}^{Close}\right),  $$

where *t* either refers to the closing price on the first day of trading or the closing price one month after IPO, and *k* is equal to either 91, 183 or 366 days, depending on the used definition of the long-term. The two starting dates are considered to control for a potential immediate drop in price after the first day of trading.

For the Google search volume (usually referred to as *GSV* in the literature), we utilize the daily statistics provided by the Google Trends database. Google provides *GSV* as a normalized measure of online searches and as such, the value shows the changes in proportion of the given searched term in the whole sample of searches rather than dynamics of the searches themselves. Again in correspondence to the standards in the literature, we utilize the abnormal *GSV* usually labeled as *ASVI* (Abnormal Search Volume Index) which is defined as a logarithmic deviation of the actual logarithmic *GSV* from the logarithm of the median *GSV* over a specific time period. In our application, we use the median period of the last 26 trading days^a^. Therefore, if we refer to *GSV* in the text, it represents the original Google search queries, and *ASVI* stands for the logarithmic deviation from the 26-day median value.

#### 2.1.2 Dataset

We use the firm database of emerging growth IPOs (Kenney and Patton 2013) to identify firms going public between years 2004 and 2010. The database contains a complete list of emerging growth firms going public at the US exchanges between 1990 and 2010. We limit ourselves to the period between 2004 and 2010 due to the Google searches data span which starts in 2004. The complete list of variables can be found in the respective guide written by its authors^b^.

The database excludes the following types of firms and filings from the Thomson Financial Venture Expert, SDC data and other comprehensive lists of IPOs: mutual funds, real estate investment trusts (REITs), asset acquisition or blank check companies, foreign F-1 filers, and all spin-offs and other firms that are not true emerging growth firms (Da et al. 2011).

We use all the companies included in the Kenney-Patton database that went public between years 2004 and 2010, with the exception of the unit offerings and one firm that went public on the OTC (over the counter) market. This encompasses 547 companies in total. For the identification of relevant search queries, we follow the steps of Bank *et al.* (2011) and Vlastakis and Markellos (2012). The complete list of search terms is available from the authors upon request. Out of the 547 companies, the daily data were available only for 75 of them^c^. Using the daily rather than weekly data thus comes at a cost. However, the frequency of missing values is comparable with other studies (Da et al. 2011) considering the additional information value provided by higher frequency of the series.

The IPOs database (Kenney and Patton 2013) does not contain data on the post-IPO performance. Therefore, the financial data on the first day closing prices come from *SCOOP Track Record from 2000 to Present* IPO database^d^, which has been checked against data from Yahoo! Finance, Google Finance, NASDAQ web site database and IPO news coverage. For the long-term performance, the data availability is also poor as some of the companies have been already acquired, merged or delisted, and therefore do not appear in the freely available databases anymore. Thus, we utilize the Quantshare Trading Software^e^, or more specifically the *Historical EOD data Downloader for Delisted/Bankrupt Stocks* plug-in^f^ for such stocks. When possible, these have been again checked against the SCOOP Track Record database, Yahoo! Finance, Google Finance, NASDAQ web site and news coverage for comparison. The final IPO data set contains search volumes and stock prices for 75 firms, even though long-term cumulative returns are available only for 62 firms. Table 1 lists and describes all variables used in the computational sections for the IPO data set.

**Table 1:** **Used variables and their definition**

**Variable**	**Definition**
*GSV*	Original Google search volume for given keyword
*ASVI*	Logarithm of *GSV* for given day minus the logarithm of median *GSV* during previous 26 days
*IR*	Log initial return of IPO calculated from the offering price to the first day closing price
*L* *R* ^(1)^	Log cumulative return calculated from the first day closing price to the closing price one year after IPO
*L* *R* ^(2)^	Log cumulative return calculated from the first day closing price to the closing price half a year after IPO
*L* *R* ^(3)^	Log cumulative return calculated from the first day closing price to the closing price a quarter after IPO
*L* *R* ^(4)^	Log cumulative return calculated from the closing price one month after IPO to the closing price one year after IPO
*L* *R* ^(5)^	Log cumulative return calculated from the closing price one month after IPO to the closing price half a year after IPO
*T* *D*_*i*_	True discount of IPO defined as in Ma and Tsai (2002). $TD=\frac {P_{e}-P_{o}}{P_{o}}$ where *P*_*o*_ is the offering price and *P*_*e*_ is the so-called equilibrium price – in our case the average price between *t*+150 and *t*+180, where *t* is the IPO date
*M* *R*_*i*_	Market reaction to IPO defined as in Ma and Tsai (2002). $MR=\frac {P_{m}-P_{e}}{P_{o}}$ where *P*_*o*_ is the offering price, *P*_*m*_ is the first day closing price and *P*_*e*_ is the so-called equilibrium price - in this case the average price between *t*+150 and *t*+180, where *t* is the IPO date
*POSSENT*	Dummy variable that takes value of one if the level of *SENTIMENT* exceeds the third quartile, and zero otherwise
*NOSENT*	Dummy variable that takes value of one if the level of *SENTIMENT* is between the first and the third quartile, and zero otherwise
*NEGSENT*	Dummy variable that takes value of one if the level of *SENTIMENT* is below the first quartile, and zero otherwise
*A* *S* *V* *I*×*S* *E* *N* *T*	*ASVI* and *SENTIMENT* interaction variable
*A* *S* *V* *I* ^*POSSENT*^	Interaction variable that takes value of *ASVI* if the level of *SENTIMENT* exceeds the third quartile, and zero otherwise
*A* *S* *V* *I* ^*NOSENT*^	Interaction variable that takes value of *ASVI* if the level of *SENTIMENT* is between the first and the third quartile, and zero otherwise
*A* *S* *V* *I* ^*NEGSENT*^	Interaction variable that takes value of *ASVI* if the level of *SENTIMENT* is below the first quartile, and zero otherwise
*A* *S* *V* *I*×*I* *R*	*ASVI* and *IR* interaction variable
*O* *f* *f* *e* *r* *i* *n* *g* *s* *i* *z* *e*	Log size of the offering measured in the US dollars
*NYSE*	Dummy variable that take one if the offering emits its shares at NYSE and zero if it emits its shares at NASDAQ
*Crisis*	Dummy variable that takes value of one for days in interval 〈3, *D* *e* *c* *e* *m* *b* *e* *r* 2007; 30, *J* *u* *n* *e* 2009〉, and zero otherwise
*Sentiment*	Monthly time-varying aggregate market sentiment orthogonalized with respect to a set of macroeconomic conditions developed by Baker and Wurgler (2006)
△*S* *e* *n* *t* *i* *m* *e* *n* *t*	Month on month difference in time-varying aggregate market sentiment orthogonalized with respect to a set of macroeconomic conditions developed by Baker and Wurgler (2006)

### 2.2 Regression analysis

The IPO regressions are all estimated by the cross-sectional ordinary least squares (OLS) procedure. We perform a widely applied methodology to test for the OLS assumptions. First, the presence of heteroskedasticity is tested by the Breusch and Pagan test (1979) and the White test (1980). No severe heteroskedasticity is detected in the sample. However, if any of the tests suggest presence of mild heteroskedasticity, White’s heteroskedasticity consistent standard errors are used (White 1980). Second, the existence of multicollinearity is tested by the variance inflation factors. Last, the normality of residuals is tested by the Shapiro-Wilk test (Shapiro and Wilk 1964). When the Shapiro-Wilk test suggests the residuals are non-normally distributed, we use bootstrapping (1000 replications) procedure to estimate the *t*-statistics and *p*-values.

## Results

We study 75 initial public offerings, which took place in the USA between 2004 and 2010, based on the Kenney-Patton database (Kenney and Patton 2013). As a measure of investor attention, we utilize Google searches provided by the Google Trends database^g^. For more details about the dataset selection process and variable construction, please refer to the Methods/Data section. Basic descriptives statistics are provided in Table 2. The initial returns are on average positive, positively skewed and fat-tailed, strongly rejecting normality. The long-term returns show opposite statistics with a negative mean and longer left tail, again strongly rejecting normality. These findings are independent of the long-term return definition. We thus observe a reversal between initial and long-term returns, at least on average. More detailed examination is provided in the following text. The true discount is on average positive and the market reaction is very close to zero. And the offering size varies strongly across the examined IPOs.

**Table 2:** **Descriptive statistics**

	***IR***	***L*** ***R*** ^**(1)**^	***L*** ***R*** ^**(2)**^	***L*** ***R*** ^**(3)**^	***L*** ***R*** ^**(4)**^	***L*** ***R*** ^**(5)**^	***T*** ***D***_***i***_	***M*** ***R***_***i***_	**Sentiment**	**Offering size**
Mean	0.1674	-0.1493	-0.1009	-0.1047	-0.0847	-0.1338	0.1823	0.0147	-0.0377	45940289
Standard deviation	0.1755	0.8209	0.5972	0.5117	0.5831	0.7996	0.4774	0.4787	0.1868	52693289
Skewness	0.6898	-1.5550	-3.3834	-4.2012	-3.8768	-1.7060	0.6830	-0.5256	0.2299	3.7917
Excess kurtosis	1.3477	4.2849	18.8820	25.2421	22.6924	5.4199	1.4454	1.3726	0.1345	21.2899
Jarque-Bera test	11.6233	72.4156	1039	1828	1485	106	10.2183	7.7218	0.7171	1596
*p*-value	<0.01	<0.01	<0.01	<0.01	<0.01	<0.01	<0.01	<0.05	>0.10	<0.01

**Figure 1:**
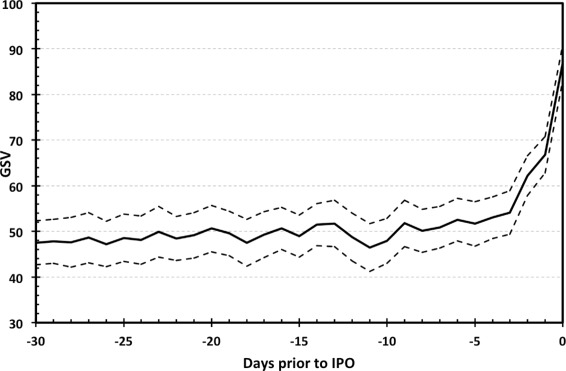
**Increase in investor attention prior to IPO.** The vertical axis shows the average *GSV* for the analyzed sample, dashed lines represent the 95% confidence intervals. The horizontal axis shows the number of days left to IPO.

To illustrate the importance and potential usefulness of the Google searches in the IPO setting, we start with the average dynamics of the Google Search Volume (*GSV*) before IPO takes place. Figure 1 shows the average *GSV* for the studied 75 IPOs together with the 95% confidence intervals. The dynamics up to 30 days before IPO takes place is presented. We can see that the investor attention starts rising around 5 five days prior to IPO. This strongly justifies using daily data in the IPO analysis contrary to the standardly used weekly frequency. We now focus on the two IPO stylized facts – the high initial returns and the long-term underperformance.

### 3.1 Initial returns

We analyze whether the search volume brings some information or predictive power regarding the IPO first day return, which is labelled as *IR* in the following text. The investor sentiment theory (Aggarwal et al. 2002; Demers and Lewellen 2003; Loughran and Ritter 2002) states that the initial returns tend to be higher in periods of positive sentiment. Da *et al.* (2011) argue that the investor sentiment attention are closely related for retail investors as these are prone to sentiment while attention is a necessary condition for sentiment. Nonetheless, we measure the effect of both attention (firm specific) and sentiment (market level) on the first day returns.

Before proceeding to the regression analysis, we examine the relationship between the initial returns and investor attention on a basic level. We divide the firms from the sample into three groups based on their *ASVI* values (Abnormal Search Volume Index, see the Methods/Data section for more details) prior IPO – high, medium and low attention groups – based on quantiles. The results show that the high attention group’s average initial return is 22.85*%*, while the low attention group’s initial return only equals to 12.23*%*. The difference is statistically significant at 5*%*. Thus, the first look at the data suggests that investor attention, very likely, drives the first day returns up.

Relationship between the initial return *IR* and the investor attention *ASVI* is estimated via the following model

(3) $$ {IR}_{i}=\beta_{0}+\beta_{1}{ASVI}_{i}+\sum\limits_{j}\gamma_{j}{CON}_{j,i}+\varepsilon_{i}  $$

in order to estimate how an increase in attention prior IPO influences the size of the initial return in more detail. *CON* represents a set of control variables, specifically the offering size and investor sentiment (both in levels and a change to previous month). Table 3 provides the results. Column (1) shows that the steeper the increase in attention prior to the IPO is, the higher the corresponding initial returns are. The effect is highly significant and has a notable size – a standard deviation increment in *ASVI* leads to an increase in initial return by a magnitude of 41.4*%* of its standard deviation.

**Table 3:** **IPO first-day return and ASVI**

	**(1)**	**(2)**	**(3)**	**(4)**	**(5)**	**(6)**	**(7)**	**(8)**	**(9)**	**(10)**	**(11)**	**(12)**	**(13)**
*A* *S* *V* *I*_*i*_	0.414***				0.400***	0.437***	0.397***	0.404***	0.357***				
	(3.311)				(3.138)	(3.768)	(3.169)	(3.217)	(3.084)				
*O* *f* *f* *e* *r* *i* *n* *g* *s* *i* *z* *e*_*i*_		-0.119			-0.094		-0.015	-0.130	-0.068				
		(-1.090)			(-0.929)		(-0.153)	(-1.269)	(-0.685)				
*S* *e* *n* *t* *i* *m* *e* *n* *t*_*i*_			0.112			0.003		-0.020			-0.039		
			(0.876)			(0.025)		(-0.177)			(-0.242)		
△*S* *e* *n* *t* *i* *m* *e* *n* *t*_*i*_				0.044					0.082			-0.037	
				(0.345)					(0.711)			(-0.291)	
${ASVI}_{i}^{POSSENT,st}$										0.297**	0.275**	0.268**	0.344***
										(2.600)	(2.034)	(2.276)	(2.815)
${ASVI}_{i}^{NEGSENT,st}$										0.163	0.152	0.136	0.280
										(1.231)	(1.253)	(1.260)	(1.539)
${ASVI}_{i}^{NOSENT,st}$													0.268
													(1.365)
*Constant*	0.003	-0.045	-0.044	0.023	-0.065	-0.034	-0.019	-0.073	-0.089	-0.011	-0.048	-0.028	0.064
	(0.026)	(-0.443)	(-0.398)	(0.203)	(-0.681)	(-0.369)	(-0.190)	(-0.763)	(-0.936)	(-0.109)	(-0.484)	(-0.275)	(0.574)
*N*	70	72	67	70	67	65	67	63	66	69	68	67	66

The IPO first day return *IR*_*i*_ is the dependent variable in each regression. *IR*_*i*_ and the independent variables are defined in Table 1. *, **, and *** represent significance at the 10%, 5%, and 1% level, respectively, standard errors are shown in the parentheses. *N* is the number of observations.

Columns (2) to (9), which display the results of the robust-check regressions, suggest that neither the offering size nor the investor sentiment (both in levels and changes from the previous month level) are able to predict initial returns. The insignificance of the offering size variable is in contradiction with results of Da *et al.* (2011), who used IPO data set with 185 firms that went public between 2004 and 2007. Thus it seems that the offering size effect over the initial return largely depends on a selected sample of firms as well as quality and availability of the Google data, which are increasing in time. The authors have also found the change in investor sentiment modestly significant (at 10% level), which is not significant in our results either.

To test the sentiment hypothesis, we construct dummy variables for positive, normal and negative values of sentiment and use them in the interaction with *ASVI* in regressions (10) to (13) in Table 3. The results show that attention significantly increases initial returns only in positive sentiment periods. For the negative and normal sentiment times, attention boosts initial returns as well, albeit the effect is not significant. Nevertheless, the difference between the three coefficients in (13) is insignificant when tested by *F*-test. In addition, regressions (11) and (12) show that the results are robust if one controls for the original sentiment measures.

### 3.2 Long-term returns

We now approach the second stylized fact about IPOs – the long-term underpricing of the IPO firms compared to their already traded peers. The sentiment-based hypothesis regarding high first day returns works well with the subsequent long-term underperformance. The investors’ overoptimism about the offering may lead to overly escalated initial returns, which should be followed by a price reversion towards the fundamental value afterwards, i.e. the long-term underperformance (Ljungqvist et al. 2006; Ritter and Welch 2002).

**Figure 2:**
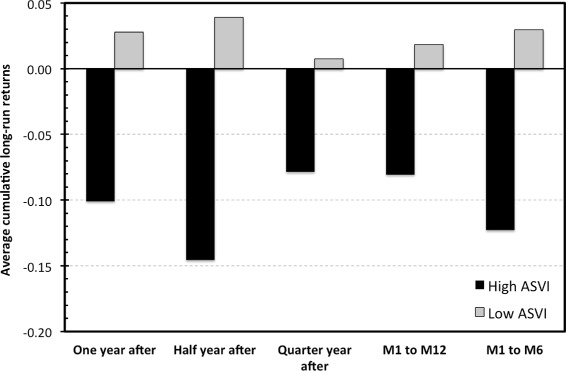
**Long-term cumulative returns for the low and high attention IPOs.** The average cumulative log-returns: first day closing price to the (1) closing price one year, (2) half a year (3) and 91 days after IPO; and the closing price one month after IPO to (4) the closing price one year (5) and half a year after IPO.

We consider five different time horizons for long-term performance for which the cumulative log-returns are calculated: first day closing price to the (1) closing price one year, (2) half a year (3) and quarter of the year after the IPO; and the closing price one month after the IPO to (4) the closing price one year (5) and half a year after the IPO. Such an approach is used to avoid coincidental results based on a randomly selected period marked as the long-term. Figure 2 provides an overview of the cumulative returns over the five specified horizons for the low and high attention IPOs. It seems that, with an exception of the shortest horizon, the high attention IPOs clearly under-perform the low attention ones in the long-term. Thus, the first results are in line with the findings of Da *et al.* (2011) and the attention/sentiment based theory on IPOs.

We proceed by regressing the long-term returns on the abnormal search volume on the IPO date. Table 4 compares the predictive power of *ASVI* over the long-term cumulative returns (LR) for the five defined periods. The results provide only weak evidence for the ability of *ASVI* to forecast the negative long-run returns. For the half-year horizon (measured both from the opening day (2) and one month after IPO (5)), *ASVI* negatively correlates with the LR returns. Nevertheless, we see no significant effect on the one year (1, 4) or quarter of the year (3) cumulative returns regardless all coefficients being negative in sign.

**Table 4:** **IPO long-term performance and ASVI**

	**(1)**	**(2)**	**(3)**	**(4)**	**(5)**
*A* *S* *V* *I*_*i*_	-0.171	-0.204*	-0.0662	-0.190	-0.187**
	(-1.19)	(-1.97)	(-0.63)	(-1.29)	(-2.15)
*Constant*	0.0292	0.0711	0.102	0.0265	0.0775
	(0.22)	(0.84)	(1.30)	(0.19)	(1.00)
*N*	59	60	59	59	60

The long-term performance *LR*_*i*_ and the independent variables are defined in Table 1. The columns show over which period the cumulative return is calculated: first day closing price to the (1) closing price one year, (2) half a year (3) and 91 days after IPO; and the closing price one month after IPO to (4) the closing price one year (5) and half a year after IPO. *, **, and *** represent significance at the 10%, 5%, and 1% level, respectively, standard errors are shown in the parentheses. *N* is the number of observations.

Da *et al.* (2011) construct an interaction variable between *ASVI* and the initial return (*ASVI*×*IR*) as the high initial return of the IPOs that also experience increases in retail investor attention should be partly driven by the price pressure and hence revert in the long-term. We follow their procedure and regress the cumulative long-term returns on initial returns and the interaction variables. Table 5 shows that there is, as expected, a higher price reversion for the IPOs that experienced high initial returns (1-5), albeit the effect is significant only for cumulative returns measured from one month after IPO. The performance of the interaction variable (5-10) matches the findings of Da *et al.* (2011) – it is obvious that the high attention IPOs with high first day return experience a severe price reversion in the long-term. The effect is significant for all considered horizons with the exception of the quarter of the year horizon measured from the offering day. It seems, and the results from the other regressions support this claim, that a quarter of the year horizon is too short for the prices to revert to their long-term level.

**Table 5:** **IPO long-term performance, ASVI and initial returns**

	**(1)**	**(2)**	**(3)**	**(4)**	**(5)**	**(1)**	**(2)**	**(3)**	**(4)**	**(5)**
*I* *R*_*i*_	-0.143	-0.053	-0.020	-0.221**	-0.162**					
	(-1.263)	(-0.566)	(-0.237)	(-2.083)	(-2.052)					
*A* *S* *V* *I*×*I* *R*_*i*_						-0.387*	-0.317**	0.112	-0.411**	-0.293**
						(-1.94)	(-2.19)	(1.07)	(-2.47)	(-2.16)
*Constant*	0.221**	0.218***	0.197***	0.195**	0.200***	0.0185	0.0768	0.176***	-0.0229	0.104
	(2.477)	(3.094)	(3.048)	(2.245)	(3.185)	(0.14)	(0.93)	(2.76)	(-0.18)	(1.35)
*N*	56	56	57	57	57	58	59	58	60	60

The cumulative long-term return *LR*_*i*_ is the dependent variable in each regression. *LR*_*i*_ and the independent variables are defined in Table 1. The columns show over which period the cumulative return is calculated: first day closing price to the (1) closing price one year, (2) half a year (3) and 91 days after IPO; and the closing price one month after IPO to (4) the closing price one year (5) and half a year after IPO. *, **, and *** represent significance at the 10%, 5%, and 1% level, respectively, standard errors are shown in the parentheses. *N* is the number of observations.

We further employ the sentiment (dummy) interaction with *ASVI* to account for the effect of attention on the long-term returns in positive, medium and negative sentiment periods. We regress the long-term returns on *ASVI* in different sentiment periods. Results are provided in Table 6. Interestingly, only the IPOs that went public in high sentiment periods and get abnormal attention show the price reversion in the long-term. Nevertheless, also sentiment itself is able to predict the long-term reversal, albeit for fewer horizons and with lower significance.

**Table 6:** **IPO long-term performance, ASVI and sentiment**

	**(1)**	**(2)**	**(3)**	**(4)**	**(5)**	**(1)**	**(2)**	**(3)**	**(4)**	**(5)**
${ASVI}_{i}^{POSSENT,st}$	-0.388**	-0.206**	0.007	-0.326***	-0.094					
	(-2.340)	(-2.300)	(0.067)	(-2.932)	(-0.813)					
${ASVI}_{i}^{NOSENT,st}$	-0.033	-0.019	0.247	-0.008	-0.019					
	(-0.189)	(-0.099)	(1.127)	(-0.048)	(-0.101)					
${ASVI}_{i}^{NEGSENT,st}$	0.056	-0.052	0.034	0.085	0.013					
	(0.449)	(-0.573)	(0.410)	(0.729)	(0.134)					
*P* *O* *S* *S* *E* *N* *T*_*i*_						-0.414**	-0.058	-0.003	-0.423**	-0.053
						(-2.066)	(-0.273)	(-0.014)	(-2.116)	(-0.252)
*N* *O* *S* *E* *N* *T*_*i*_						0.189	-0.073	-0.044	0.163	-0.118
						(0.900)	(-0.329)	(-0.200)	(0.780)	(-0.538)
*N* *E* *G* *S* *E* *N* *T*_*i*_						0.309	0.158	0.055	0.350	0.206
						(1.363)	(0.665)	(0.232)	(1.550)	(0.866)
*Constant*	0.031	0.111	0.208**	0.056	0.160*					
	(0.281)	(1.232)	(2.302)	(0.552)	(1.823)					
*N*	54	57	56	55	55	62	62	62	62	62

The cumulative long-term return *LR*_*i*_is the dependent variable in each regression. *LR*_*i*_ and the independent variables are defined in Table 1. The columns show over which period the cumulative return is calculated: first day closing price to the (1) closing price one year, (2) half a year (3) and 91 days after IPO; and the closing price one month after IPO to (4) the closing price one year (5) and half a year after IPO. *, **, and *** represent significance at the 10%, 5%, and 1% level, respectively, standard errors are shown in the parentheses. *N* is the number of observations.

### 3.3 Initial returns versus underpricing

The terms “initial return” and “underpricing” are usually used interchangeably. However, Ma and Tsai (2002) argue that under the sentiment hypothesis, the interchangeability is not correct. According to their definition, the initial return has two components – true discount (*TD*) and market reaction (*MR*) – and it is split in the following way

(4) $$ IR=TD+MR=\frac{P_{m}-P_{o}}{P_{o}}=\frac{P_{e}-P_{o}}{P_{o}}+\frac{P_{m}-P_{e}}{P_{o}}  $$

where *P*_*m*_ is the first day closing price, *P*_*o*_ is the offer price and *P*_*e*_ is the equilibrium (fundamental) market price. In the previous section, we have shown that the price revision and reversion for the high attention IPOs happens approximately half a year after the offering. Moreover, if return variance is calculated for 30-day periods up to one year after IPO, the lowest variance corresponds to a horizon between 150 and 180 days after emission. Therefore, we use the average price between *t*+150 and *t*+180, where *t* is the IPO date, as an estimate for *P*_*e*_. Note that any estimate of the fundamental price is rather arbitrary so that other definitions are indeed feasible.

**Figure 3:**
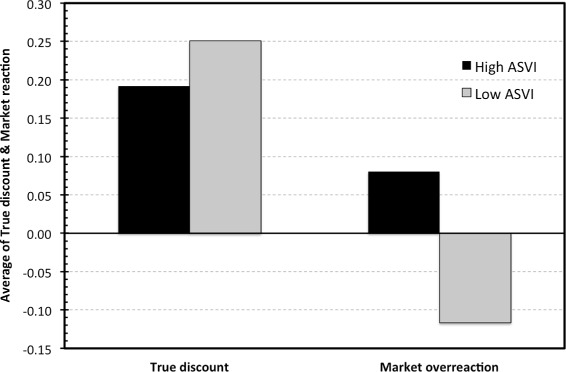
**True discount and market overreaction for the low and high attention IPOs.**

According to the authors (Ma and Tsai 2002), the positive values of *MR* suggest that investors overreact, while the negative values suggest investors’ under-reaction. The true discount, on the other hand, corresponds to the actual underpricing. Thus, we use this setting to confirm the results that *ASVI*, especially if combined with positive sentiment on the market, drives the investor overreaction. In contrast, we expect that *ASVI* should not possess any significant information about the underpricing term *TD*. To see whether such expectations are valid, we calculate mean *TD* and *MR* for the high and low attention IPOs. Figure 3 displays the comparison. As expected, the true discount does not seem to be influenced by attention. Conversely, the market reaction and attention devoted to IPO show strong interdependence.

The relationship is majorly confirmed by the regression results. We regress *TD* and *MR* on attention measured by *ASVI*, on the *ASVI* interaction with the initial return, and on the attention-sentiment interaction variables. Results are presented in Table 7. On the one hand, it can be observed that no attention-based variable predicts the underpricing term. On the other hand, market seems to overreact on the high attention IPOs, albeit the effect is significant only at 10%. The effect is more pronounced if we take into account the interaction with initial return, which is logical as *MR* is one of the two terms which the initial return consists of (the evidence is thus stronger against *ASVI* and *TD* interdependence, as the interaction term is insignificant in *TD*). Surprisingly, we see only an insignificant effect of the sentiment interaction variables and the market reaction. While the coefficient is positive for attention in positive sentiment periods, it is insignificant (albeit on the edge of 10% significance). Even more surprising is the positive coefficient for the attention in negative sentiment periods, as one would expect this term to be negative. It suggests that investors overreact to IPOs also in low sentiment period and that it is the attention that drives the overreaction and not sentiment. This is confirmed by regression (8), which shows that sentiment is not able to predict the market reaction on its own. The insignificance is indisputable in this case.

**Table 7:** **Ma-Tsai model and ASVI**

	**(1)**	**(2)**	**(3)**	**(4)**	**(5)**	**(6)**	**(7)**	**(8)**
	***T*** ***D***_***i***_	***M*** ***R***_***i***_	***T*** ***D***_***i***_	***M*** ***R***_***i***_	***T*** ***D***_***i***_	***M*** ***R***_***i***_	***T*** ***D***_***i***_	***M*** ***R***_***i***_
*A* *S* *V* *I*_*i*_	0.00754	0.221*						
	(0.06)	(1.88)						
*A* *S* *V* *I*×*I* *R*_*i*_			0.109	0.428*				
			(0.62)	(1.86)				
${ASVI}_{i}^{POSSENT,st}$					-0.130	0.252		
					(-0.717)	(1.641)		
${ASVI}_{i}^{NOSENT,st}$					-0.081	0.083		
					(-0.494)	(0.482)		
${ASVI}_{i}^{NEGSENT,st}$					0.025	0.153		
					(0.214)	(1.136)		
*P* *O* *S* *S* *E* *N* *T*_*i*_							0.098	-0.013
							(0.464)	(-0.063)
*N* *O* *S* *E* *N* *T*_*i*_							-0.130	-0.042
							(-0.590)	(-0.190)
*N* *E* *G* *S* *E* *N* *T*_*i*_							0.027	0.066
							(0.114)	(0.276)
*Constant*	-0.0451	0.0946	-0.0406	0.0389	-0.067	0.051		
	(-0.39)	(0.83)	(-0.39)	(0.31)	(-0.590)	(0.457)		
*N*	58	56	56	57	56	55	62	62

The dependent variables are true discount *TD*_*i*_ and market reaction *MR*_*i*_ as defined by Ma & Tsai (2002). *TD*_*i*_, *MR*_*i*_ and independent variables are defined in Table 1. *, **, and *** represent significance at the 10%, 5%, and 1% level, respectively, standard errors are shown in the parentheses. *N* is the number of observations.

## Discussion

We confirm that initial returns are higher for the IPOs that receive above average attention. However, we argue that the effect is significantly present only for the firms going public in the positive sentiment periods. In addition, since the daily data are used, we are able to demonstrate that Google search volume is capable of forecasting the initial returns within a few days horizon.

Contrary to Da *et al.* (2011), we observe a weak evidence of Google data ability to forecast (with negative sign) the long-term cumulative returns. Nevertheless, in line with the authors, we show that the high attention IPOs leaving a lot of money on the table experience a price reversal in long-term. In correspondence with the initial returns results, the long-term cumulative returns seem to be inversely proportional to the IPO investor attention only for firms that emitted shares during the positive sentiment periods. The findings correspond to predictions of Derrien (2005) claiming that it is the overoptimistic investors who leave the money on the table rather than the issuing firms.

Finally, we test Google search volume in the setting of the model proposed by Ma and Tsai (2002), which questions the interchangeability of terms initial return and underpricing. The results suggest that the Google search volume is able to predict one part of initial returns – the market overreaction to the offering –, while the other – the true IPO discount (i.e. the underpricing) – is unpredictable by Google data, which is in fact expected.

## Endnotes

^a^ The median period of 26 trading days is chosen as it is close to a trading month and such choice delivers the best results. However, it needs to be noted that the results do not change qualitatively for the median periods between 20 and 30 trading days.

^b^ The guide is available at http://hcd.ucdavis.edu/faculty/webpages/kenney/misc/Firm_IPO_Database_Guide.pdf.

^c^ Google Trends system allows to download daily series for a period of up to three months. For our given dataset, we have selected a three-month period covering the IPO date for each company.

^d^ The database is available at https://www.iposcoop.com/index.php?option=com_trackrecord&Itemid=200.

^e^ The software is available at http://www.quantshare.com/.

^f^ The plug-in is available at http://www.quantshare.com/item-1270-historical-eod-data-downloader-for-delisted-bankrupt-stocks.

^g^ Freely available at http://trends.google.com/. Google data are registered trademarks of Google Inc., used with permission.
